# Health interoperability across phenotypes of family physician practices

**DOI:** 10.1093/jamia/ocaf178

**Published:** 2025-10-27

**Authors:** Jordan Everson, Catherine Strawley

**Affiliations:** Assistant Secretary for Technology Policy and Office of the National Coordinator for Health Information Technology, US Department of Health and Human Services, Washington, DC 20201, United States; Department of Family Medicine, Georgetown University School of Medicine, Washington, DC 20007, United States; Assistant Secretary for Technology Policy and Office of the National Coordinator for Health Information Technology, US Department of Health and Human Services, Washington, DC 20201, United States

**Keywords:** physicians, family medicine, interoperability, latent class analysis, health information exchange

## Abstract

**Objectives:**

To inform initiatives to improve the interoperability of healthcare data, we described the experience of distinct phenotypes of physicians when obtaining information from outside sources.

**Materials and Methods:**

A total of 6175 family physicians across the United States responded to information technology questions on the 2022 and 2023 American Board of Family Medicine Continuous Certification Questionnaire (100% response rate). Latent class analysis grouped physicians by individual and practice characteristics and then compared reported experience with interoperability.

**Results:**

A 4-class model (“Safety Net,” “Health System,” “Independent Practice,” and “Large Practice”) best fit. Health system and large practice physicians (predominately Epic users) were more likely to report information was integrated in their Electronic Health Record (EHR) than independent practice physicians (38% and 40%, respectively, compared to 24%), and to report that information from organizations using the same EHR was usable (52% and 51%, respectively, compared to 25%). Safety net physicians were least likely to report that information from outside organizations was usable (17% compared to 23% of independent physicians). Between 42% and 50% of each phenotype reported commonly encountering external records with a large volume of low-value information.

**Discussion:**

Independent practice and safety net physicians reported worse experience in some dimensions of interoperability, likely driven by differences in access to information from organizations using the same EHR. Many other challenges were consistent across physician phenotypes.

**Conclusion:**

Initiatives to improve interoperability among physicians may be most effective if targeted at independent practices and safety net practices; however, broad improvements will be necessary to address similar challenges across phenotypes.

## Background and significance

For over 2 decades, federal and state policy, as well as private efforts, have focused on encouraging seamless interoperable exchange of patient health information between healthcare delivery organizations. Despite these efforts, interoperability from the physician’s perspective continues to mature unevenly.^1^^,^^2^ Interoperability remains the primary improvement to the Electronic Health Record (EHR) requested by primary care physicians, and physician satisfaction with access to information from outside organizations is generally low and varies widely.^1^^,^^3^ There is also evidence that shows many physicians do not use interoperable tools that could potentially address these challenges.^4^ Effective policy and practice interventions could be assisted by evidence that informs targeted efforts to improve adoption and use of tools, reduce barriers to exchange, and improve satisfaction.

Prior studies have used regression-based techniques to identify the independent relationship between specific physician, practice, and EHR system characteristics and physicians’ experience with interoperability.^5–7^ A challenge with this approach is that these variables are often highly correlated and likely practically inseparable. For example, external forces drive physician employment decisions, and organizational factors drive the selection of EHR system so that separately considering organizational employment and EHR selection may not be valuable.^1^^,^^5^^,^^8^ An alternative approach is to identify groups of physicians based on the co-occurrence of these characteristics. This approach would allow for tracking physician experience across coherent phenotypes, facilitate targeted interventions toward those groups with worse interoperability experience, and more easily identify and apply best practices learned from those with more positive experiences.

Identification of phenotypes could inform ongoing policymaking and the potential value of initiatives that would target specific groups of clinicians. As two examples, the Centers for Medicare & Medicaid Services’ Merit-based Incentive Payment System (MIPS) Promoting Interoperability Component, provide financial incentives and disincentives related to engagement in interoperability but specifically excludes small practices from those incentives.^9^ Thus, further analysis could be warranted to determine effective ways to improve small practices’ interoperability. Similarly, the Health Resources and Services Administration (HRSA) specifically supports safety net clinics through grantmaking to organizations that provide technical assistance to safety net providers.^10–12^ Evidence that safety net providers are on equal footing with or ahead of other organizations with respect to interoperability would demonstrate the effectiveness of that support. In contrast, evidence on how they lag behind could guide further investment or alternative strategies.^13^ Limited variation in interoperability across groups—including those targeted and not targeted by public incentive programs—would indicate the extent to which the certification of health information technology and EHRs through the Office of the National Coordinator for Health IT (ONC) Health IT Certification Program has effectively established a nationwide baseline for interoperable exchange.

## Objective

We used national survey data on family physicians to define phenotypes of primary care physicians to better describe their experience with interoperability. We focused on primary care because of the importance of interoperability to their role as care coordinators and in facilitating continuity of care. We sought to define phenotypes of these physicians based on their demographics and practice characteristics. We then described the interoperability experience of these physicians in terms of their use of interoperable tools to obtain information from other organizations, the barriers they report to doing so, and their satisfaction with electronic access to information from other organizations.

## Materials and methods

### Data

Data from the 2022 and 2023 American Board of Family Medicine (ABFM) Continuous Certification Questionnaire (CCQ) were used to conduct this analysis. The Office of the Assistant Secretary for Technology Policy/Office of the National Coordinator for Health IT (ASTP/ONC), ABFM, and the University of California, San Francisco have been collaborating since 2021 to generate survey questions relevant to physicians’ use of interoperable exchange technologies and satisfaction with health information technology (health IT). The CCQ is a requirement for family physicians renewing their board certification, and thus, these questions receive a 100% response rate. The 2022 data reflect the survey responses of 4247 providers, and the 2023 data include responses from 8390 physicians providing outpatient care. We combined these 2 years into one dataset for analysis. Both years include questions relating to the use of technologies to view exchanged data; the extent of challenges to data availability, quality, and completeness; and ease of (or satisfaction with) access to and usability of external clinical information. These questions were asked of a random sample of half of all respondents.

### Physician phenotype measures

To define phenotypes of physicians, we drew demographic and practice characteristics from the CCQ to capture site specialty, practice ownership (1) Academic health center/faculty practice; (2) safety net (Federally Qualified Health Center, Rural Health Clinic [federally qualified] Government clinic, non-federal); (3) independently owned medical practice; (4) other (Managed care/Health Maintenance Organization [HMO], Indian Health Service, Federal clinic, Workplace clinic, “Other”), site size, physician age and sex, provision of value-based care, the portion of patients that are considered vulnerable, primary EHR system used (with some EHR vendor categories combined due to small sample sizes as detailed in Appendix SA2), years of experience using primary EHR, and overall EHR satisfaction. See Appendix Table SA2—Part 1 for more details.

### Outcome measures

Among the phenotypes identified based on these characteristics, we assessed differences in the use of tools for information exchange, barriers to exchanging and receiving information, and interoperability satisfaction to understand physicians’ overall experiences with interoperability. Use of interoperable tools was measured using 3 questions designed to directly capture physicians’ firsthand experience by asking, “When you access clinical information about your patients from outside your organization (eg, referrals, consult notes, discharge summaries, patient records), how often is it” (1) available as a scanned document, (2) in an electronic portal, and (3) within the EHR in an integrated format. Barriers were assessed by physician responses to questions regarding the extent to which external records are not available, information within external records is missing or unavailable, external information is not integrated within the EHR, and external records contain a high volume of low-value information. Finally, interoperability satisfaction was evaluated using responses from questions relating to the ease of using external clinical information for patient care, finding specific information from external clinical records, and using external clinical information from both the same and different EHR vendors. Finally, we included a previously defined satisfaction index variable, reflecting 10 questions asking providers to rate their current satisfaction with their ability to access different types of external patient information electronically.^13^ See Appendix Table SA2—Part 2 for more details about these items.

### Analysis

To define physician phenotypes, we developed a latent class model based on respondents’ individual and practice characteristics as well as the EHR used and their satisfaction with the EHR using the ‘poLCA’ package in R (4.2.2). Because we had information on individual and practice characteristics from all respondents, we identified classes using the full set of survey respondents, including those who did not provide information on their experience with interoperability. We believe this approach allows for the identification of stable classes based on statistical relationships present in the largest available set of respondents. We selected latent class models, rather than k-means or similar cluster analysis, because latent class models are better suited to ordinal survey data. To identify the best-fitting number of classes, we calculated the Akaike Information Criterion (AIC) and Bayesian Information Criterion (BIC) values for each model. The number of classes for the final model was selected by examining AIC and BICs and identifying the point at wherein additional classes led only to marginal improvements in these criteria (see Appendix Figure SA1). For the selected 4-class model, posterior probabilities for class assignment were extracted from the model. We selected a probability threshold of .5 to ensure respondents were not assigned to multiple classes and to limit the number of respondents not assigned to a class. We then examined local independence, an assumption of latent class analysis, by examining Cramer’s V for each pair of covariates within each class. We describe results in the appendix (Appendix Figures SA2–A7).

We next generated descriptive statistics of the independent variables used to generate the latent class model, both for the overall analytic sample and each of the classes. We considered each class as defining a phenotype of physicians and assigned a descriptive label to each phenotype based on the characteristics that were furthest from the population average for each phenotype. We subsequently calculated descriptive statistics for the interoperability experience variables within each class to determine how these experiences vary by phenotype. We compared differences across phenotypes using Student’s *t*-test. Appendix Table SA2 presents the survey questions used and recoding performed to generate analytic variables.

## Results

The final sample used to inform phenotype assignment consisted of 12 350 physician survey responses. Most of these physicians (78%, Table 1) reported working at a site with fewer than 20 providers and with a “family medicine only or primary care specialty mix” (75%). Respondents reported working at a variety of care sites, most frequently “hospital/health system-owned medical practice” (36%) (Table 1). More than two-thirds (69%) reported providing value-based care, and a minority (22%) of providers indicated that more than half of their patients were part of a vulnerable group. About half of the respondents were under the age of 50; similarly, approximately half identified as male. The most commonly reported primary EHR developer was Epic, used by 39% of respondents.

**Table 1.** Descriptive statistics of model training variables by phenotype assignment.

Variable of interest	Overall		Phenotype 1		Phenotype 2		Phenotype 3		Phenotype 4			
Independent variables used to train the 4-phenotype model	Complete analytic sample (*N* = 12 350)		Independent practice providers (*N* = 3866)		Health system providers (*N* = 3661)		Safety net providers (*N* = 2284)		Large practice providers (*N* = 2172)		Unassigned (*N* = 367)	
**Site specialty**												
Family medicine only or primary care specialty mix	78%	9589	89%	3440	93%	3423	71%	1622	38%	820	77%	284
Multiple specialties (not only primary care)	22%	2761	11%	426	7%	238	29%	662	62%	1352	23%	83
**Practice ownership**												
Academic health center/faculty practice	7%	901	0%	0	3%	116	12%	285	19%	415	23%	85
Safety net	15%	1853	4%	173	1%	24	72%	1639	0%	0	5%	17
Hospital/health system-owned medical practice	36%	4471	3%	128	87%	3187	2%	56	45%	979	33%	121
Independently owned medical practice	29%	3545	81%	3136	4%	162	0%	0	11%	236	3%	11
Other^a^	13%	1580	11%	429	5%	172	13%	304	25%	542	36%	133
**Site size**												
1-5 Providers	43%	5283	73%	2818	55%	2010	17%	381	0%	0	20%	74
6-20 Providers	32%	3967	19%	728	45%	1651	49%	1109	11%	247	63%	232
>20 Providers	25%	3100	8%	320	0%	0	35%	794	89%	1925	17%	61
**Age**												
<50	46%	5643	36%	1399	50%	1822	53%	1204	48%	1039	49%	179
50+	54%	6707	64%	2467	50%	1839	47%	1080	52%	1133	51%	188
**Sex**												
Female	47%	5757	40%	1539	48%	1745	56%	1282	47%	1010	49%	181
Male	52%	6449	59%	2289	52%	1886	42%	960	52%	1137	48%	177
Prefer not to answer	1%	144	1%	38	1%	30	2%	42	1%	25	2%	9
**Provide value-based care**												
I don’t know	18%	2183	15%	584	15%	538	23%	525	20%	430	29%	106
No	13%	1658	31%	1189	3%	120	10%	232	3%	74	12%	43
Yes	69%	8509	54%	2093	82%	3003	67%	1527	77%	1668	59%	218
**Percentage of vulnerable patients**												
<10%	36%	4431	49%	1911	39%	1436	11%	248	34%	732	28%	104
10%-49%	43%	5256	38%	1473	54%	1961	23%	522	51%	1110	52%	190
>50%	22%	2663	12%	482	7%	264	66%	1514	15%	330	20%	73
**Primary EHR used**												
Allscripts	5%	599	6%	248	4%	145	2%	46	6%	134	7%	26
athenahealth	9%	1147	14%	529	6%	225	11%	256	4%	88	13%	49
Cerner	7%	904	0%	14	13%	463	12%	266	6%	137	7%	24
eClinical works	11%	1354	23%	879	3%	126	12%	284	1%	29	10%	36
Epic	39%	4817	1%	37	69%	2533	21%	478	76%	1658	30%	111
NextGen	4%	506	5%	186	1%	46	8%	193	3%	62	5%	19
Other	23%	2798	49%	1883	3%	111	30%	677	2%	43	23%	84
Unknown	2%	225	2%	90	0%	12	4%	84	1%	21	5%	18
**Years of experience with primary EHR**												
<1	5%	622	5%	201	5%	183	9%	195	1%	30	4%	13
1-5	42%	5147	42%	1635	45%	1645	49%	1109	28%	611	40%	147
6-14	46%	5626	43%	1655	46%	1689	38%	879	56%	1217	51%	186
15+	8%	955	10%	375	4%	144	4%	101	14%	314	6%	21
**EHR satisfaction**												
Very dissatisfied	10%	1256	10%	375	8%	311	15%	346	8%	171	14%	53
Somewhat dissatisfied	17%	2054	17%	653	15%	541	23%	535	12%	268	16%	57
Neither satisfied nor dissatisfied	9%	994	8%	318	7%	252	11%	252	6%	138	9%	34
Somewhat satisfied	39%	4824	40%	1539	39%	1439	34%	778	42%	908	44%	160
Very satisfied	25%	3114	24%	944	30%	1107	14%	328	31%	674	17%	61
Not applicable	1%	108	1%	37	0%	11	2%	45	1%	13	1%	2

a Other includes Managed care/HMO, Indian Health Service, federal, workplace clinic, “Other”. Chi-squared tests indicated that all characteristics were not independent of class at *P* < .001.

The 4 identified phenotypes varied substantially across demographic and practice characteristics (Table 1). Phenotype 1, which includes 3866 physicians, represents providers who primarily work in an independently owned medical practice (81%) and use an EHR other than Epic. This phenotype was labeled “Independent Practice Physicians.”

Phenotype 2, which includes 3661 physicians, represents providers who primarily work at a hospital or health system-owned medical practice (87%) and use Epic as their primary EHR (69%). This phenotype was labeled “Health System Physicians.”

Phenotype 3, which included 2284 physicians, is predominantly composed of providers who primarily work at a safety net site (72%), have a high portion of vulnerable patients (66% in phenotype 3 reported >50% of patients were from a vulnerable population), and were less satisfied with their EHRs (23% “somewhat dissatisfied” and 15% “very dissatisfied”). This phenotype was labeled “Safety Net Physicians.”

Phenotype 4, which includes 2172 physicians, represents providers who primarily work in large practice settings (89% in this phenotype report working in a practice with 20 or more providers) and use Epic (76%). Sixty-two percent of physicians in this phenotype practiced in a multispecialty practice compared to 22% overall. This phenotype was labeled “Large Practice Physicians.” Three hundred and sixty-seven physicians were not assigned to a phenotype.

**Figure 1.**
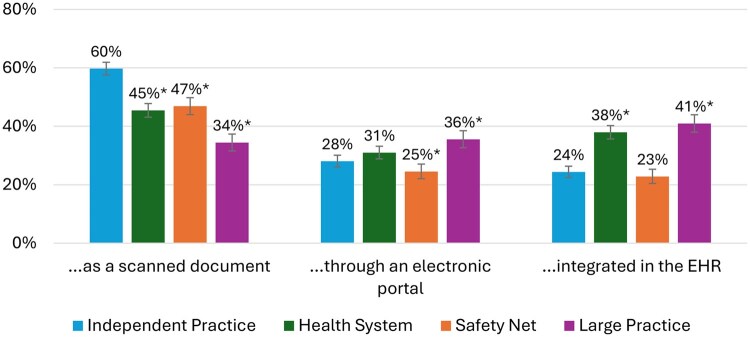
Percent of physicians in each phenotype that Report Often Accessing Information from Outside Organizations…. “Don’t know” response options were included in the denominators of the presented statistics. *Statistically significantly different from the reference group (independent practice providers) with a *P*-value of <.05. Error bars indicate 95% confidence intervals. The question stem is “When you access clinical information about your patients from outside your organization (eg, referrals, consult notes, discharge summaries, patient records), how often is it…”.

Fifty percent of survey respondents received questions related to their experience with interoperability. Across phenotypes, we observed variation in the use of different tools to access information from outside organizations (Figure 1). Independent Practice Physicians (24.4%) reported that information from outside organizations that they used was often available and integrated in the EHR. In comparison, health system physicians more frequently indicated that information from outside organization that they used was “often” integrated (24.4% vs 37.9%) (difference = 13.6; 95% CI, 10.6-16.5; *P* < .001) as did large practice physicians (40.9%) (difference = 15.5; 95% CI, 13.1-20.1; *P* < .001). Independent Practice Physicians and Safety Net Physicians reported that information from outside organizations was “often” integrated in the EHR at similar rates (24.4% vs 22.8%) (difference = −1.6; 95% CI of difference, −4.6 to 1.4; *P* = .31).

**Figure 2.**
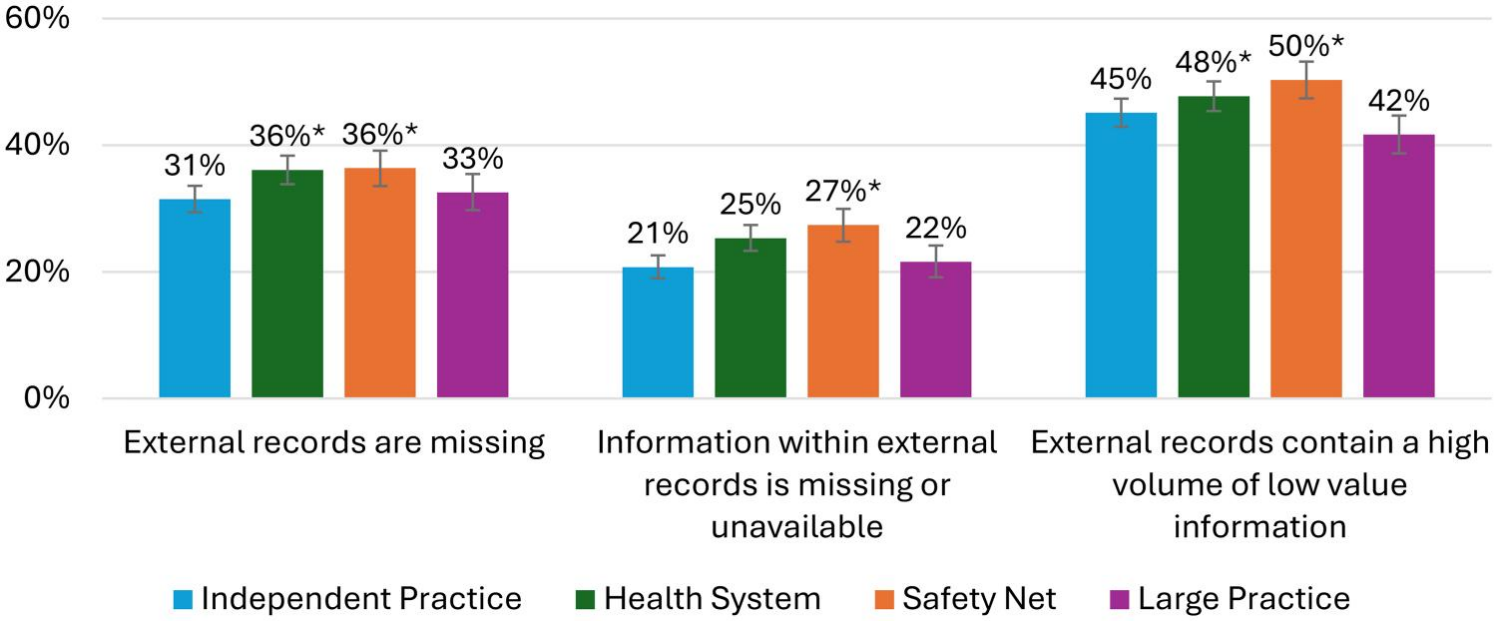
Physician reports of interoperability barriers experienced “to a great extent” by the physician phenotype. *Statistically significantly different from the reference group (independent practice providers) with a *P*-value of <.05. “Don’t know” and “Not Applicable” response options were included in the denominators of the presented statistics.

Differences in the experience of barriers to obtaining information were smaller across phenotypes (Figure 2). Independent Practice Physicians (31.5%) reported experiencing that external records were missing to a great extent. Safety Net Physicians (36.4%) (difference = 4.9; 95% CI, 1.5-8.3; *P* = .005) and Health System Physicians (36.1%) (difference = 4.6; 95% CI, 1.6-7.6; *P* = .003) were significantly more likely to report that external records were missing to a great extent. The difference between Independent Practice and Large Practice (32.6%) physicians was smaller (difference = 1.08; 95% CI, −4.6 to 2.4; *P* = .54). Despite these observed differences, across all 4 phenotypes, almost all physicians reported experiencing these 3 barriers either “to some extent” or “to a great extent.” See Appendix Table SA4 for additional data.

Compared to Independent Practice Physicians, Safety Net Physicians less frequently reported that it was “very” easy to use external clinical information for patient care (Table 2; 17.3% vs 25.5%) (difference = −8.2; 95% CI, −11.1 to −5.3; *P* < .001) and to find specific information from external clinical records (14.9% vs 23.2%) (difference = −8.2; 95% CI, −11.0 to −5.5; *P* < .001). Mean overall interoperability satisfaction scores were higher for health system (11.4) and Large Practice Physicians (11.3) compared to Independent Practice Physicians (10.2; difference = 1.2; 95% CI of difference, 0.85-1.57; *P* < .001 and difference = 1.2; 95% CI of difference, −1.6 to −0.8; *P* < .001, respectively) but lower among Safety Net Physicians (9.2; difference = −1.0; 95% CI of difference, −1.4 to −0.55; *P* < .001).

**Table 2.** Distribution of satisfaction variable values by phenotype assignment among 50% of sample receiving question on interoperability experience.

	Overall analytic sample (*N* = 6175)	Phenotype 1	Phenotype 2	Phenotype 3	Phenotype 4	Unassigned (*N* = 187)
		Independent practice physicians (*N* = 1957)	Health system physicians (*N* = 1785)	Safety net physicians (*N* = 1171)	Large practice physicians (*N* = 1075)	

Interoperability satisfaction						
	%	%	%	%	%	%
**Ease of using external clinical information for patient care**						
Very easy	23%	25%	25%	17%	24%	19%
Somewhat easy	66%	64%	65%	66%	68%	68%
Not at all easy	8%	8%	8%	12%	6%	8%
Don’t know	2%	2%	1%	3%	1%	3%
Not applicable	1%	1%	1%	2%	1%	1%
**Ease of finding specific information from external clinical records**						
Very easy	20%	23%	18%	15%	20%	20%
Somewhat easy	61%	55%	66%	57%	67%	59%
Not at all easy	15%	15%	13%	21%	10%	18%
Don’t know	2%	3%	1%	4%	2%	3%
Not applicable	2%	3%	1%	3%	1%	1%
**Ease of using external clinical information from the same EHR vendor**						
Very easy	38%	25%	52%	25%	51%	40%
Somewhat easy	33%	32%	34%	30%	34%	30%
Not at all easy	13%	18%	7%	20%	7%	15%
Don’t know	9%	11%	5%	12%	6%	10%
Not applicable	8%	13%	2%	13%	2%	5%
**Ease of using external clinical information from a different EHR vendor**						
Very easy	7%	10%	5%	6%	5%	5%
Somewhat easy	49%	48%	52%	43%	50%	51%
Not at all easy	33%	28%	36%	35%	35%	33%
Don’t know	8%	8%	6%	10%	8%	9%
Not applicable	4%	5%	2%	5%	2%	3%
**Satisfaction index**						
25th percentile	8	7	9	6	9	7
Median	10	10	10	10	10	10
Mean	10.53	10.2	11.4	9.2	11.3	9.9
75th percentile	14	14	16	12	16	13

These questions were posed to only half of all respondents, *n* = 6175. All response options, including Don’t know and Not Applicable, were retained in the denominator for each presented percent. Chi-squared tests indicated that interoperability experiences were not independent of class at *P* < .001.

Relative to Independent Practice Physicians (24.9%), Health System Physicians (52.2%) (difference = 27.2 p.p.; 95% CI, 24.2-30.2; *P* < .001) and Large Practice Physicians (51.1%) (difference = 26.1 p.p.; 95% CI, 22.6-29.7; *P* < .001) were substantially more likely to report that it was “very” easy to use external clinical information from a provider who uses the same EHR vendor. In contrast, Independent Practice Physicians reported that it was “very” easy to use external clinical information from a provider who uses a different EHR vendor at the highest rates (9.9%), with Safety Net Physicians (6.5%) (difference = −3.4; 95% CI, −5.4 to −1.4; *P* < .001), Health System Physicians (4.5%) (difference = −5.4; 95% CI, −7.0 to −3.7; *P* < .001), and Large Practice Physicians (5.2%) (difference = −4.7; 95% CI, −6.5 to −2.8; *P* < .001) reporting this level of ease at significantly lower rates.

## Discussion

Latent class analysis facilitated the identification of 4 recognizable phenotypes of family physicians, and there were important distinctions in interoperability experience across these 4 groups. Differences are primarily related to their ability to access information integrated into their EHR and their satisfaction accessing information from organizations using the same or different EHR. Despite some notable differences, many dimensions of family physicians’ experience with interoperability, especially related to the frequency of experiencing specific barriers or frictions, were similar across phenotypes, highlighting widespread challenges to seamlessly accessing data from other organizations to support high-quality, coordinated care.

The identified phenotypes demonstrate the close relationship between practice characteristics, demographics of patients served, and the EHR system. These close relationships are a challenge for observational studies seeking to identify independent relationships between practice characteristics, vulnerable patient populations, EHR vendor used, EHR satisfaction, experience with interoperability, and other variables of interest, such as EHR satisfaction and burnout.^5^^,^^14^ This challenge can lead to the suppression of important relationships because variables included in a regression model lie on the causal chain or an exaggeration of associations when important confounders are not included in the model. For instance, one recent study found no relationship between treating Medicaid patients and health information exchange (HIE) participation, but a negative correlation may have been suppressed by including practice size in the model if practices that accept Medicaid on average were smaller than those not accepting Medicaid, and larger size was associated with HIE participation.^8^ On the contrary, the association may have been biased downwards if practices treating Medicaid generally selected an EHR vendor that was less likely to connect them to HIE because that model did not include an EHR vendor. A different study reported a small difference in interoperability satisfaction between physicians in independent practices and physicians in health systems in a regression model that also included EHR vendor, which had a large association with satisfaction. This is challenging to interpret because physicians in independent practice less often select market-leading vendors and inclusion of vendor likely changed the observed association.^5^

To illustrate this, we created a logistic regression model with the outcome “Often” having information available integrated in the EHR and predictors comprised of the variables used to determine class membership (Appendix Table S5). In this model, there is no statistically significant difference in integrated information between hospital/health system-employed physicians and physicians in independent practice, likely because correlated variables are also related to integration, suppressing the association between practice ownership and integrated information, which is clear in the phenotype approach. Relative to these studies and approaches, the approach taken here to identifying classes has the practical advantage of defining distinct groups that might be targeted by policymakers or entrepreneurial initiatives to improve interoperability and highlighting some important differences in how they experience interoperability.

One plausible interpretation for this study’s findings—and in particular the finding that very few Health System and Large Practice Physicians (and overall only 6% of respondents) stated it was easy to use information from outside organizations using different EHRs and—is that even well incentivized or motivated large health systems and health IT developers have relatively little ability to improve the experience of interoperability for family physicians within their organization because that interoperability depends on the actions of other actors across the healthcare delivery system who share data with that organization. A second interpretation is that existing structures and incentives designed to support interoperability are only partially effective for all parties, except those using an EHR from the same vendor built on the same fundamental data structure. Either interpretation highlights the need for cross-industry collaboration and policymaking to address this apparent collective action failure. Policies authorized by the 21st Century Cures Act in 2016 and implemented through regulatory and programmatic actions may collectively improve interoperability.^15^ These include requirements for health IT developers to adopt HL7 Fast Healthcare Interoperability Resources (FHIR^®^)^16^ and to support the United States Core Data for Interoperability (USCDI)^17^; implementation of the Trusted Exchange Framework and Common Agreement (TEFCA),^18^ which is intended to connect health information networks to facilitate nationwide exchange; and adherence to the information blocking regulations, so that electronic health information can be accessed, exchanged, and used to the maximum extent possible.

Many of these policies are at different stages of maturity, such that their impacts will need to be iteratively studied to better understand their effectiveness. One potential concern is that policies that incentivize adoption of FHIR, participation in TEFCA, and adherence to information blocking do not apply directly to certain cohorts of the healthcare continuum, including physicians in small practices and many safety net practices below thresholds for eligibility in the MIPS, let alone long-term care providers, behavioral health care providers, and other organizations. Further, gaps remain in how health IT developers and their healthcare organization users implement the technology and how it performs in real-world settings. Variation in technical and human workflows may affect consistent delivery of high-quality data and adherence to standards, as adopted by HHS.^19–21^ Renewed policy that focuses on support for Independent Practice and Safety Net providers may be essential to improve their experience of interoperability and the experience of physicians in Large Practices and Health Systems due to the interconnected nature of interoperable exchange.

Finally, while our data focused on the experience of family medicine physicians, we believe these findings are likely to generalize to other specialties for two reasons. First, the practice characteristics used to characterize phenotypes are not specialty-specific, and so similar structures may well exist among other specialties. Second, other work across specialties has found broadly similar trends in experience with interoperability.^3^^,^^4^ In contrast, we suspect that other settings—including long-term care and some behavioral health providers—that experience different market pressures, use substantially different EHRs, and were not eligible for Meaningful Use incentives, may follow different trends.

### Limitations

This work is cross-sectional in nature and does not establish causality between the collection of physician and setting-level characteristics and the interoperability tools, barriers, and satisfaction variables evaluated. In particular, we cannot and do not seek to determine a causal order between practice characteristics (eg, practice size causing EHR vendor selection) but rather treat these variables as tied-up and descriptive of phenotypes of physicians. Relatedly, we do not test all relationships between interoperability experience and physician characteristics. Specifically, we cannot examine the relationship between EHR training, experience, or expectations and the subjective measures of interoperability reported here. A limitation of the data used to generate our latent class model was that it did not include whether physicians’ practices were connected to a health information exchange organization (HIO), and thus, this concept was not accounted for in the model. It is possible that HIO participation could be an important factor driving differences in the ease of use of external clinical information, as many HIOs make data accessible to participants in a standardized format. Further, we were not able to delineate whether physicians work in a geographic location in which most providers use the same EHR vendor, which could facilitate more seamless exchange (depending on the capabilities of the EHR) and lead to fewer experiences with barriers to interoperability.

## Conclusion

Distinct phenotypes of physicians emerged in this survey of more than 10 000 family physicians. Family physicians in health systems and large practices using Epic reported greater access to information from outside organizations integrated in their EHR, especially from other Epic sites. But physicians across phenotypes had similarly modest overall satisfaction with obtaining information from outside organizations and reported high rates of friction, indicating broad challenges to the effective exchange of information across the delivery system.

## Supplementary Material

ocaf178_Supplementary_Data

## Data Availability

Data is available from the American Board of Family Medicine (ABFM). The ABFM Research Department conducts research and partners on projects related to the environment in which family physicians deliver healthcare. Proposals are vetted by a research governance committee to determine alignment with ABFM objectives. Interested researchers can submit a proposal subject to approval by the ABFM to https://www.theabfm.org/research/external-collaborations/

## References

[1] Everson J , Hendrix N, Phillips RL, Adler-Milstein J, Bazemore A, Patel V. Primary care physicians’ satisfaction with interoperable health information technology. JAMA Netw Open. 2024;7:e243793. 38530309 10.1001/jamanetworkopen.2024.3793 PMC10966410

[2] Walker DM , Tarver WL, Jonnalagadda P, Ranbom L, Ford EW, Rahurkar S. Perspectives on challenges and opportunities for interoperability: findings from key informant interviews with stakeholders in Ohio. JMIR Med Inform. 2023;11:e43848. 36826979 10.2196/43848 PMC10007006

[3] Anderson J , Rowley T. *EHR Interoperability 2024: Clinician Needs Still Not Being Met*. KLAS Research; 2024. Accessed October 13, 2025. https://klasresearch.com/archcollaborative/report/ehr-interoperability-2024/604

[4] Office of the National Coordinator for Health Information Technology. *Electronic health information exchange by office-based physicians, Health IT Quick Stat #63*. 2023. Accessed September 1, 2025. https://www.healthit.gov/data/quickstats/electronic-health-information-exchange-office-based-physicians

[5] Everson J , Barker W, Patel V. Electronic health record developer market segmentation contributes to divide in physician interoperable exchange. J Am Med Inform Assoc. 2022;29:1200-1207. 35442438 10.1093/jamia/ocac056 PMC9196705

[6] Apathy NC , Vest JR, Adler-Milstein J, Blackburn J, Dixon BE, Harle CA. Practice and market factors associated with provider volume of health information exchange. J Am Med Inform Assoc. 2021;28:1451-1460. 33674854 10.1093/jamia/ocab024 PMC8279783

[7] Esmaeilzadeh P. Identification of barriers affecting the use of health information exchange (HIE) in clinicians’ practices: an empirical study in the United States. Technol Soc. 2022;70:102007.

[8] McCullough M , Stecher J. C. Associations between physician practice models and health information exchange. Am J Manag Care. 2023;29:27-34. 36716152 10.37765/ajmc.2023.89301

[9] Centers for Medicare & Medicaid Services. Quality payment program special statuses. Accessed September 1, 2025. https://qpp.cms.gov/mips/special-statuses? py=2024#small-practice

[10] Administration HRaS. HCCN cooperative agreements. 2023. Accessed September 1, 2025. https://bphc.hrsa.gov/funding/funding-opportunities/health-center-controlled-networks-hccn

[11] Centers for Medicare & Medicaid Services. Promoting interoperability. 2024. Accessed September 1, 2025. https://qpp.cms.gov/mips/promoting-interoperability

[12] Strategy to support health information technology among HRSA’s safety-net providers. *Federal Register*. 2008;73:4584.

[13] Cross DA , Stevens MA, Spivack S, Murray GF, Rodriguez H, Lewis VA. A survey of information exchange and use of other Health IT in primary care settings: capabilities in and outside of the Safety Net. Medical Care. 2022;60:140-148. 35030563 10.1097/MLR.0000000000001673 PMC8966676

[14] Rotenstein LS , Apathy N, Landon B, Bates DW. Assessment of satisfaction with the electronic health record among physicians in physician-owned vs non–physician-owned practices. JAMA Netw Open. 2022;5:e228301-e. 35446397 10.1001/jamanetworkopen.2022.8301 PMC9024386

[15] 21st Century Cures Act, 114-255, Vol 162 (2016) (Year).

[16] HL7 FHIR Overview. Accessed September 1, 2025. https://www.hl7.org/fhir/overview.html

[17] Office of the Assistant Secretary for Technology and Policy. *United States Core Data for Interoperability (USCDI)*. 2022. Accessed September 1, 2025. https://www.healthit.gov/isa/united-states-core-data-interoperability-uscdi#uscdi-v2

[18] Tripathi M , Yeager M. *TEFCA Live! The future of network interoperability is here*. Health Affairs Forefront. 2023.

[19] Assistant Secretary for Technology and Policy. *Real world testing*. Accessed September 1, 2025. https://www.healthit.gov/topic/certification-ehrs/real-world-testing

[20] Assistant Secretary for Technology and Policy. *Inferno on HealthIT.gov*. Accessed September 1, 2025. https://inferno.healthit.gov/

[21] Assistant Secretary for Technology and Policy. *Insights Condition*. 2024. Accessed September 1, 2025. https://www.healthit.gov/topic/certification-health-it/insights-condition

