# Supplementary material for: Health interoperability across phenotypes of family physician practices
Source: J Am Med Inform Assoc. 2025 Oct 27;33(2):434–41. doi: 10.1093/jamia/ocaf178 (PMC12844596; doi:10.1093/jamia/ocaf178)
Supplement: ocaf178_Supplementary_Data [file ocaf178_supplementary_data.docx]

**Appendix**

**Appendix Table 1. Independent Variable Descriptive Statistics – ABFM 2022 vs. 2023 Survey**

|  | **2022**  **(N=4,154)** | | **2023**  **(N=8,196)** | |
| --- | --- | --- | --- | --- |
| Site specialty |  |  |  |  |
| Family medicine only or primary care specialty mix | 77% | 3218 | 78% | 6371 |
| Multiple specialties (not only primary care) | 23% | 936 | 22% | 1825 |
| Main site type |  |  |  |  |
| Academic health center / faculty practice | 8% | 312 | 7% | 589 |
| Governmental | 16% | 649 | 15% | 1204 |
| Hospital / health system owned medical practice | 36% | 1479 | 37% | 2992 |
| Independently owned medical practice | 27% | 1130 | 29% | 2415 |
| Other | 14% | 584 | 12% | 996 |
| Site size |  |  |  |  |
| 1-5 Providers | 42% | 1733 | 43% | 3550 |
| 6-20 Providers | 31% | 1306 | 32% | 2661 |
| >20 Providers | 27% | 1115 | 24% | 1985 |
| Age |  |  |  |  |
| <50 | 60% | 2491 | 38% | 3152 |
| 50+ | 40% | 1663 | 62% | 5044 |
| Sex |  |  |  |  |
| Female | 50% | 2069 | 45% | 3688 |
| Male | 50% | 2085 | 53% | 4364 |
| Prefer not to answer |  |  | 2% | 144 |
| Provide value-based care |  |  |  |  |
| I don't know | 20% | 833 | 16% | 1350 |
| No | 13% | 526 | 14% | 1132 |
| Yes | 67% | 2795 | 70% | 5714 |
| Percentage of vulnerable patients |  |  |  |  |
| <10% | 34% | 1394 | 37% | 3037 |
| 10-49% | 43% | 1781 | 42% | 3475 |
| >50% | 24% | 979 | 21% | 1684 |
| Primary EHR used |  |  |  |  |
| Allscripts | 5% | 210 | 5% | 389 |
| athenahealth | 9% | 380 | 9% | 767 |
| Cerner | 7% | 307 | 7% | 597 |
| eClinical Works | 11% | 448 | 11% | 906 |
| Epic | 38% | 1570 | 40% | 3247 |
| NextGen | 4% | 178 | 4% | 328 |
| Other | 24% | 967 | 23% | 1831 |
| Unknown | 2% | 94 | 2% | 131 |
| Years of experience with primary EHR |  |  |  |  |
| <1 | 5% | 226 | 5% | 396 |
| 1-5 | 44% | 1837 | 40% | 3310 |
| 6-14 | 46% | 1913 | 45% | 3713 |
| 15+ | 4% | 178 | 9% | 777 |
| EHR satisfaction |  |  |  |  |
| Very dissatisfied | 8% | 419 | 8% | 837 |
| Somewhat dissatisfied | 17% | 344 | 17% | 650 |
| Neither satisfied nor dissatisfied | 38% | 690 | 39% | 1364 |
| Somewhat satisfied | 10% | 1595 | 10% | 3229 |
| Very satisfied | 26% | 1063 | 25% | 2051 |
| Not Applicable | 1% | 43 | 1% | 65 |
| Notes: Missing values are excluded from the denominator. | | | | |

**Appendix Table A2 – Variable Descriptions and Recoding**

Part 1: Independent Variables

| Concept | Survey Question / Variable Code |
| --- | --- |
| Physician and Practice Specialties | |
| Site specialty | Which of the following describes the physician specialty mix of your principal practice:   1. Family medicine only or primary care specialty mix 2. Multiple specialties (not only primary care) |
| Main site of care | Which of the following describes your principal practice site:   1. Academic health center / faculty practice 2. Governmental 3. Hospital / health system owned medical practice 4. Independently owned medical practice 5. Other |
| Site size | Which of the following describes your principal practice size:   1. 1-5 Providers 2. 6-20 Providers 3. >20 Providers |
| Age | Information on file with ABFM |
| Sex | Information on file with ABFM |
| Provision of value-based care | Does your organization participate in one or more value-based care initiative(s), such as a patient centered medical home, accountable care organization or pay-for-performance arrangement?   1. Yes 2. No 3. Don’t know |
| Percentage of vulnerable patients | What percentage of your patient population in your principal practice site is part of a vulnerable group (i.e. uninsured, medicaid, homeless, low income, non-english speaking, racial/ethnic minority, or otherwise traditionally underserved group)   1. <10% 2. 10-49% 3. >50% |
| Primary EHR system used* | What is the name of your current PRIMARY, outpatient EHR system? CHECK ONLY ONE BOX. IF OTHER IS CHECKED, PLEASE SPECIFY THE NAME.   1. Allscripts 2. athenahealth 3. Cerner 4. eClinical Works 5. e-MDs 6. Epic 7. Modernizing Medicine 8. NextGen 9. Practice Fusion 10. Greenway 11. Other 12. Unknown   Recoding Approach:  Due to small sample size, any records reporting “e-MDs,” “Greenway,” “Modernizing Medicine,” and “Practice Fusion” were recoded as “Other specified EHR.” |
| Years of experience using primary EHR* | How long have you used your current primary, outpatient EHR system?  Recoding Approach:  In the original data, this variable was coded as a continuous numeric variable. This was recoded as a categorical variable with the following categories:   1. 0-1 years 2. 2-5 years 3. 6-10 years 4. >10 years |
| Overall EHR satisfaction | Overall, how satisfied are you with your current primary, outpatient EHR system?   1. Very dissatisfied 2. Somewhat dissatisfied 3. Neither satisfied nor dissatisfied 4. Somewhat satisfied 5. Very satisfied 6. Not applicable |

*Indicates that variable recoding was performed.

Part 2: Dependent Variables

| Concept | Survey Question / Variable Code |
| --- | --- |
| Interoperability Tools | |
| Availability of external clinical information as a scanned document | When you access clinical information about your patients from outside your organization (e.g. referrals, consult notes, discharge summaries, patient records), how often is it available as a scanned document?   1. Never 2. Rarely 3. Sometimes 4. Often 5. Don’t Know |
| Availability of external clinical information through an electronic portal | When you access clinical information about your patients from outside your organization (e.g. referrals, consult notes, discharge summaries, patient records), how often is it in an electronic portal (e.g. to a health information exchange) separate from your EHR?   1. Never 2. Rarely 3. Sometimes 4. Often 5. Don’t Know |
| Availability of external clinical information in the EHR | When you access clinical information about your patients from outside your organization (e.g. referrals, consult notes, discharge summaries, patient records), how often is it from within your EHR in any integrated format (as opposed to a PDF)?   1. Never 2. Rarely 3. Sometimes 4. Often 5. Don’t Know |
| Barriers | |
| External records are not available | When looking for or using clinical information from outside your organization, to what extent do the following occur: Entire record is not available   1. Not at all 2. To Some Extent 3. To a Great Extent 4. Not Applicable |
| Information within external records is missing or unavailable | When looking for or using clinical information from outside your organization, to what extent do the following occur: Key information within record is missing/not available   1. Not at all 2. To Some Extent 3. To a Great Extent 4. Not Applicable |
| External information is not integrated within the EHR | When looking for or using clinical information from outside your organization, to what extent do the following occur: Information is not integrated within my EHR   1. Not at all 2. To Some Extent 3. To a Great Extent 4. Not Applicable |
| High volume of low value information in external records | When looking for or using clinical information from outside your organization, to what extent do the following occur: Difficulty finding important information due to a large amount of low-value information   1. Not at all 2. To Some Extent 3. To a Great Extent 4. Not Applicable |
| Interoperability Satisfaction | |
| Ease of using external clinical information for patient care | When you access clinical information from outside your organization (e.g. referrals, consult notes, discharge summaries, patient records) through any means (e.g. fax, phone, EHR, etc), how easy is it to use the information to effectively care for your patients?   1. Not at all 2. Somewhat 3. Very 4. Not Applicable 5. Don’t Know |
| Ease of finding specific information from external clinical records | When you access clinical information from outside your organization in your EHR, how easy is it to find specific information (e.g., medications, vitals, procedures, lab results)?   1. Not at all 2. Somewhat 3. Very 4. Not Applicable 5. Don’t Know |
| Ease of using external clinical information – same EHR vendor | How easy is it to use clinical information from clinicians outside your organization that use the same EHR Vendor?   1. Not at all 2. Somewhat 3. Very 4. Not Applicable 5. Don’t Know |
| Ease of using external clinical information – different EHR vendor | How easy is it to use clinical information from clinicians outside your organization that use a different EHR Vendor   1. Not at all 2. Somewhat 3. Very 4. Not Applicable 5. Don’t Know |
| EHR satisfaction index* | An index of EHR satisfaction (on a scale of 0-20) developed using the following question:  Rate your current satisfaction with accessing the following types of external patient information electronically:   1. Prior encounters (e.g., hospitalizations, ED visits) 2. Clinical notes 3. Labs 4. Vitals 5. Immunizations 6. Radiology reports 7. Information on preventative care (e.g., last mammography date) 8. Discharge/care summaries 9. Electronic notifications regarding patient visits to Emergency Department   Response options:   1. Don’t have/Don’t use it 2. Not at all satisfied 3. Somewhat satisfied 4. Very satisfied   The satisfaction index variable was coded according to the approach used by Everson et. al (2024). For each type of external patient information, a respondent was assigned 0 points for a response of “Don’t have/Don’t use it” or “Not at all satisfied”, 1 point for a response of “Somewhat satisfied”, and 2 points for a response of “Very satisfied.” The final satisfaction index variable value was calculated as a summation of these scores. |

*Indicates that variable recoding was performed.

**Additional Model Checks**

When fitting the latent class model on the analytic sample frame, calculated values for the Bayesian Information Criterion (BIC) and Akaike Information Criterion (AIC) began to plateau when the number of classes considered increased from 3 to 4, such that each model with an additional class (after 4 classes) improved BIC and AIC only marginally, but at the expense of a less parsimonious model (Appendix Figure A1). When considering the same set of training variables, as defined previously, BIC decreased from 244741 for the 3-class model to 242935.1 for the 4-class model but only decreased to 242076.5 for the 5-class model. Similarly, AIC decreased from 244040.2 for the 3-class model to 241998.2 for the 4-class model but only decreased to 240903.6 for the 5-class model. Based on these results, the study team selected the 4-class model for this analysis.

**Appendix Figure A1. Performance metrics by number of classes.**


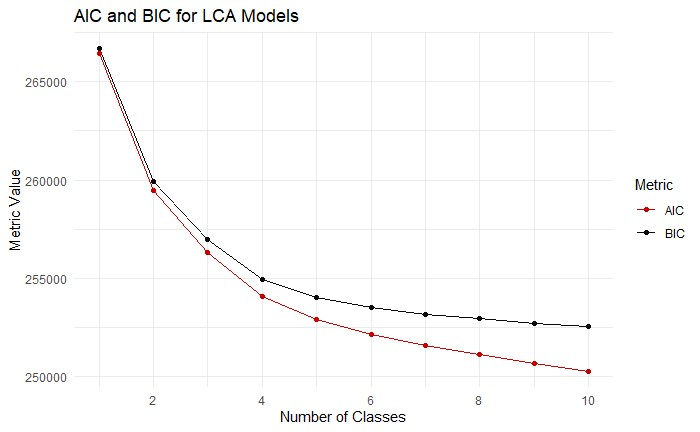


After confirming the appropriateness of the 4-class model, a probability threshold of 0.5 was selected for class assignment. At a probability threshold of 0.5, 381 responses were not assigned to one of the four classes; there was a subsequent sharp increase in the number of left out responses, which reached 779 when the probability threshold was increased to 0.55 (Appendix Figure A2). The number of left out responses was just 165 at a probability threshold of 0.45, but this led to an issue of multiple class assignment.

**Appendix Figure A2. Proportion of respondents assigned to each class, by probability threshold.**

**
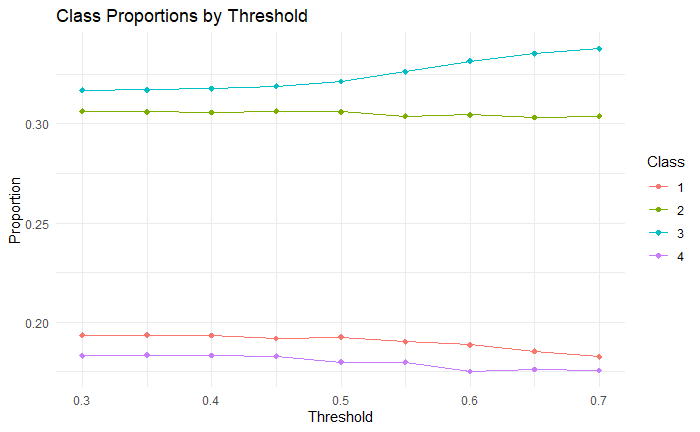
**

A sensitivity analysis demonstrated that the proportion of respondent physicians assigned to each class was similar for thresholds ranging from 0.3 to 0.5 but then began to differ slightly as the threshold was increased to 0.7 (Appendix Figure A3) Therefore, a probability threshold of 0.5 for class assignment would result in similar assignments as other lower threshold options while ensuring that the model is as representative of the overall analytic data set as possible by minimizing the number of respondents left out from class assignment. Based on these results, respondent physicians were assigned to a class if the posterior probability of being a part of that respective latent class was greater than or equal to 0.5. Notably, there was no issue with multiple class assignment at a probability threshold of 0.5.

**Appendix Figure A3. Observations left and assigned to multiple classes by probability threshold for class assignment.**


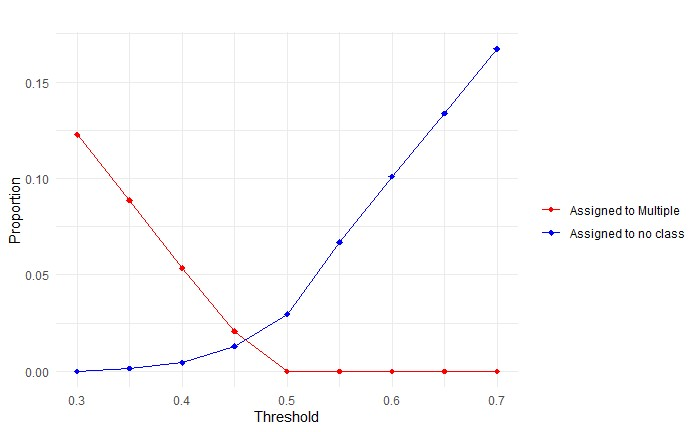


Calculations of Cramer’s V to assess the relationships between the model training variables in the four-class model confirmed that the assumption of local independence was met (Appendix Figures A4-A7). Across all classes, Cramer’s V values for all combinations of training variables were below 0.3, except for in class four, in which the Cramer’s V value representing the relationship between the site specialty and main site variables was just 0.34.

**Appendix Figure A4. Heatmap of Cramer’s V for model training variables for class 1 of the 4-class model.**


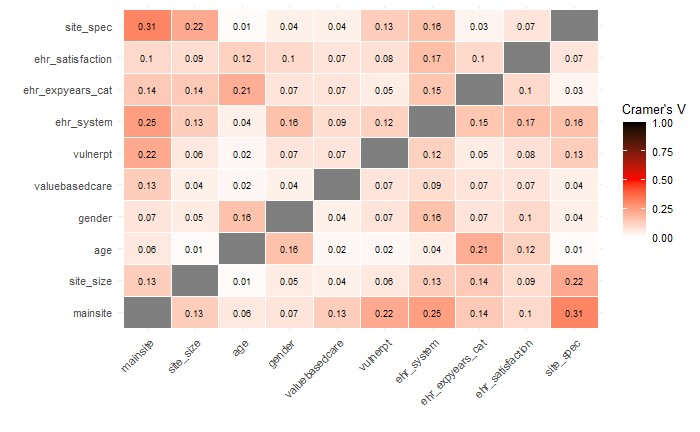


**Appendix Figure A5. Heatmap of Cramer’s V for model training variables for class 2 of the 4-class model.**
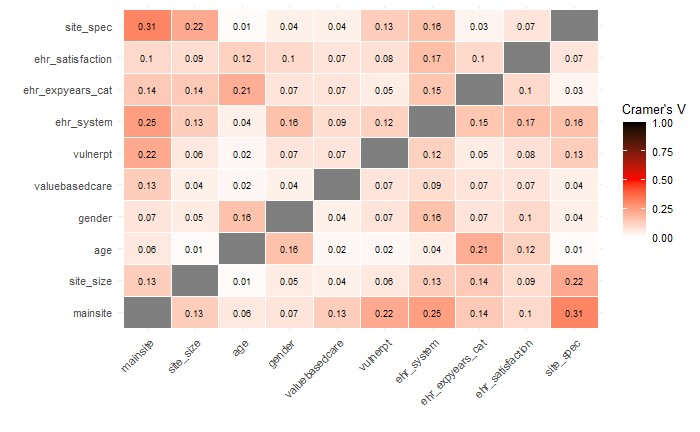


**Appendix Figure A6. Heatmap of Cramer’s V for model training variables for class 3 of the 4-class model.**


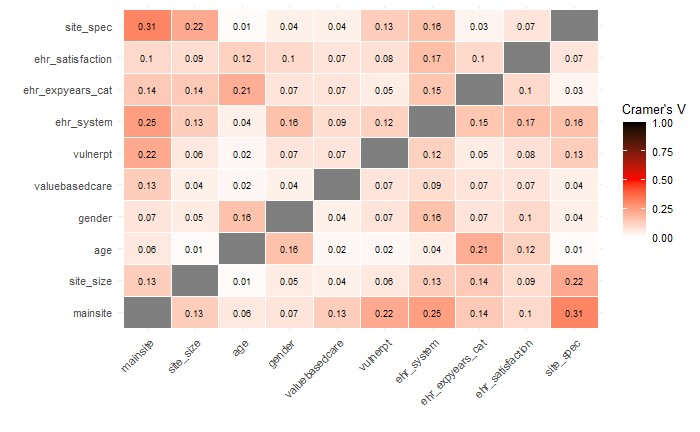


**Appendix Figure A7. Heatmap of Cramer’s V for model training variables for class 1 of the 4-class model.**
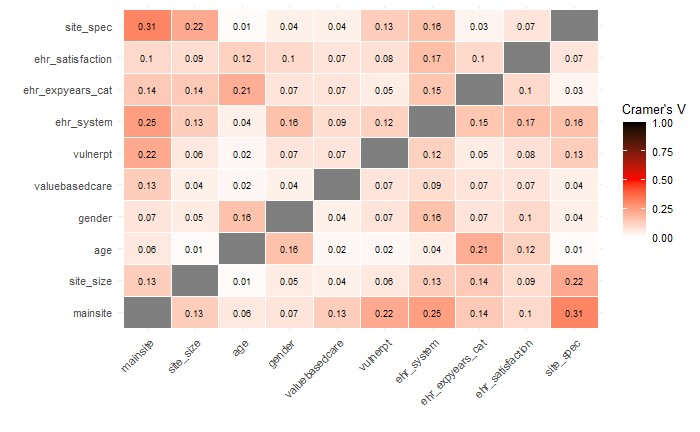


**Appendix Table A3.** Distribution of interoperability tools variable values by class assignment.

|  | **Overall Analytic Sample***  **(N=6,175)** | | **Class 1** | | **Class 1** | | **Class 2** | | **Class 3** | | **Unassigned (N=187)** | |
| --- | --- | --- | --- | --- | --- | --- | --- | --- | --- | --- | --- | --- |
|  |  |  | **Safety Net Providers (N=1,171)** | | **Health System Providers (N=1,785)** | | **Independent Practice Providers (N=1,957)** | | **Large Practice Providers (N=1,075** | |  |  |
| Interop Tools: Availability of external clinical information… | | | | | | | | | | | | |
| …as a scanned document |  |  |  |  |  |  |  |  |  |  |  |  |
| Often | 24% | 2988 | 47% | 549 | 45% | 811 | 60% | 1169 | 34% | 370 | 48% | 89 |
| Sometimes | 20% | 2422 | 40% | 463 | 45% | 811 | 29% | 568 | 48% | 515 | 35% | 65 |
| Rarely | 4% | 476 | 8% | 95 | 6% | 113 | 6% | 120 | 12% | 131 | 9% | 17 |
| Never | 1% | 125 | 2% | 22 | 1% | 19 | 2% | 47 | 3% | 29 | 4% | 8 |
| Don’t know | 1% | 164 | 4% | 42 | 2% | 31 | 3% | 53 | 3% | 30 | 4% | 8 |
| …through an electronic portal |  |  |  |  |  |  |  |  |  |  |  |  |
| Often | 15% | 1819 | 25% | 287 | 31% | 553 | 28% | 549 | 36% | 382 | 26% | 48 |
| Sometimes | 19% | 2286 | 34% | 401 | 42% | 750 | 31% | 614 | 41% | 445 | 41% | 76 |
| Rarely | 7% | 906 | 16% | 182 | 13% | 231 | 18% | 360 | 10% | 110 | 12% | 23 |
| Never | 6% | 793 | 17% | 201 | 9% | 164 | 16% | 317 | 8% | 84 | 14% | 27 |
| Don’t know | 3% | 371 | 9% | 100 | 5% | 87 | 6% | 117 | 5% | 54 | 7% | 13 |
| …in the EHR |  |  |  |  |  |  |  |  |  |  |  |  |
| Often | 16% | 1921 | 23% | 267 | 38% | 677 | 24% | 477 | 41% | 440 | 32% | 60 |
| Sometimes | 16% | 1966 | 28% | 331 | 36% | 646 | 26% | 513 | 39% | 416 | 32% | 60 |
| Rarely | 7% | 836 | 16% | 183 | 11% | 200 | 18% | 343 | 9% | 92 | 10% | 18 |
| Never | 8% | 959 | 23% | 275 | 8% | 134 | 24% | 461 | 5% | 56 | 18% | 33 |
| Don’t know | 4% | 493 | 10% | 115 | 7% | 128 | 8% | 163 | 7% | 71 | 9% | 16 |
| Notes: * These questions were posed to only half of all respondents, n=6,175. Here, the analytic sample excludes 825 records for which one or more of the variables used to generate the latent class model had a missing value, as well as respondents who were not asked the subset of questions pertaining to interoperability tool use, barriers, and interoperability satisfaction. | | | | | | | | | | | | |

**Appendix Table A4.** Distribution of barriers variable values by class assignment.

|  | **Overall Analytic Sample***  **(N=6,175)** | | **Class 1** | | **Class 2** | | **Class 3** | | **Class 4** | | **Unassigned (N=187)** | |
| --- | --- | --- | --- | --- | --- | --- | --- | --- | --- | --- | --- | --- |
|  |  |  | **Safety Net Providers (N=1,171)** | | **Health System Providers (N=1,785)** | | **Independent Practice Providers (N=1,957)** | | **Large Practice Providers (N=1,075** | |  |  |
| Barriers | | | | | | | | | | | | |
| External records are missing | % | N | % | N | % | N | % | N | % | N | % | N |
| To a Great Extent | 34% | 2094 | 36% | 426 | 36% | 644 | 31% | 616 | 33% | 350 | 31% | 58 |
| To Some Extent | 55% | 3397 | 52% | 607 | 56% | 1000 | 54% | 1059 | 59% | 629 | 55% | 102 |
| Not at all | 6% | 379 | 6% | 67 | 5% | 84 | 8% | 155 | 5% | 56 | 9% | 17 |
| Not Applicable | 5% | 305 | 6% | 71 | 3% | 57 | 6% | 127 | 4% | 40 | 5% | 10 |
| Information within external records is missing or unavailable |  |  |  |  |  |  |  |  |  |  |  |  |
| To a Great Extent | 24% | 1459 | 27% | 321 | 25% | 452 | 21% | 406 | 22% | 232 | 26% | 48 |
| To Some Extent | 64% | 3929 | 60% | 701 | 66% | 1178 | 61% | 1212 | 67% | 725 | 60% | 113 |
| Not at all | 7% | 459 | 6% | 74 | 5% | 95 | 10% | 204 | 7% | 73 | 7% | 13 |
| Not Applicable | 5% | 328 | 6% | 75 | 3% | 60 | 7% | 135 | 4% | 45 | 7% | 13 |
| External records contain a high volume of low value information |  |  |  |  |  |  |  |  |  |  |  |  |
| To a Great Extent | 46% | 2858 | 50% | 589 | 48% | 852 | 45% | 883 | 42% | 448 | 46% | 86 |
| To Some Extent | 43% | 2638 | 38% | 446 | 45% | 802 | 40% | 786 | 49% | 523 | 43% | 81 |
| Not at all | 5% | 337 | 5% | 55 | 4% | 72 | 7% | 140 | 6% | 60 | 5% | 10 |
| Not Applicable | 6% | 342 | 7% | 81 | 3% | 59 | 8% | 148 | 4% | 44 | 5% | 10 |
| Notes: These questions were posed to only half of all respondents, n=6,175. Here, the analytic sample excludes 825 records for which one or more of the variables used to generate the latent class model had a missing value, as well as respondents who were not asked the subset of questions pertaining to interoperability tool use, barriers, and interoperability satisfaction. | | | | | | | | | | | | |

**Appendix Table 5.** Logistic regression predicting Information Often Integrated into the EHR by Physician characteristics used in latent class model.

|  | Odds Ratio | 95% Confidence Interval |
| --- | --- | --- |
| Multiple specialties (not only primary care) (Ref: Family Medicine Only) | 0.968 | [0.833, 1.123] |
| **Practice ownership** |  |  |
| Academic health center / faculty practice | 0.723** | [0.567, 0.918] |
| Governmental | 0.884 | [0.721, 1.083] |
| Independently owned medical practice | 1.088 | [0.913, 1.296] |
| Other | 1.164 | [0.962, 1.408] |
| **Site size** |  |  |
| site_size1-5 Providers | 0.913 | [0.771, 1.082] |
| site_size6-20 Providers | 0.890 | [0.758, 1.046] |
| 50 or older (Ref: Under 50) | 1.284*** | [1.141, 1.447] |
| Gender (Ref: Female) |  |  |
| Male | 1.162* | [1.034, 1.306] |
| Other / prefer not to answer | 0.711 | [0.370, 1.280] |
| **Provide value-based care (Ref: Don’t Know** |  |  |
| No | 0.843 | [0.671, 1.059] |
| Yes | 1.169+ | [0.999, 1.369] |
| **Percentage of vulnerable patients (Ref: <10%)** |  |  |
| 10-49% | 0.873* | [0.767, 0.994] |
| >50% | 0.924 | [0.779, 1.096] |
| **Primary EHR used (Ref: AllScripts)** |  |  |
| athenahealth | 1.575** | [1.120, 2.236] |
| Cerner | 1.163 | [0.800, 1.699] |
| eClinical Works | 0.973 | [0.692, 1.381] |
| Epic | 2.542*** | [1.875, 3.491] |
| NextGen | 0.873 | [0.567, 1.340] |
| Other | 0.987 | [0.714, 1.380] |
| Unknown | 0.890 | [0.476, 1.603] |
| **Years of experience with primary EHR (Ref: <1)** |  |  |
| 1-5 | 0.802 | [0.606, 1.069] |
| 6-14 | 0.796 | [0.601, 1.062] |
| 15+ | 0.849 | [0.606, 1.195] |
| **EHR satisfaction (Ref: Very dissatisfied)** |  |  |
| Not Applicable | 0.447+ | [0.165, 1.022] |
| Somewhat dissatisfied | 1.044 | [0.804, 1.359] |
| Somewhat satisfied | 1.599*** | [1.275, 2.019] |
| Very dissatisfied | 0.948 | [0.709, 1.270] |
| Very satisfied | 2.501*** | [1.976, 3.183] |
| Number of Hospitals | 6175 |  |
| + p < 0.1, * p < 0.05, ** p < 0.01, *** p < 0.001 | |  |
